# Learning needs and experiences of hospital nurses in genetic medicine in a rural area of Japan: A cross-sectional questionnaire survey in Oita prefecture

**DOI:** 10.1016/j.pmedr.2025.103239

**Published:** 2025-09-12

**Authors:** Nobue Tsukatani, Yumi Shimada, Masanori Inoue, Akiko Hatanaka, Shizuyo Tominaga, Kenji Ihara

**Affiliations:** a Genetic Medical Center, Oita University Hospital, 1-1 Idaigaoka, Hasama, Yufu, Oita 879-5593, Japan; b Department of Pediatrics, Faculty of Medicine, Oita University, 1-1 Idaigaoka, Hasama, Yufu, Oita 879-5593, Japan; c Nursing Department, Oita University Hospital, 1-1 Idaigaoka, Hasama, Yufu, Oita 879-5593, Japan

**Keywords:** Genetic counseling, Genetic medicine, Cancer genomic profiling, Hospital nurses, Rural health, Nursing education, Japan

## Abstract

*Objective:* This study aimed to investigate hospital nurses' experiences in providing care to patients with genetic diseases and their families and to identify their educational needs regarding genetic medicine and genetic counseling in Oita Prefecture, a representative rural region of Japan.

*Methods:* This cross-sectional questionnaire survey was conducted at ten cancer treatment hospitals in Oita Prefecture, including two central hospitals for cancer genomic medicine and eight regional hospitals, from October 11, 2021 to January 20, 2022. Of 1881 invited nurses, 1001 valid responses were analyzed. Data were collected using an anonymous self-administered questionnaire covering demographics, understanding of genetic terms, care experiences, and learning needs.

*Results:* The respondents showed limited understanding of clinical genetics terminology, with fewer than 5 % having received genetics-related training. Only 8.6 % had experience in caring for patients and their families concerns about genetic diseases, and over 80 % of those felt inadequate. More than 70 % expressed a strong desire to learn about genetic medicine.

*Conclusions:* Hospital nurses in rural Japan have significant learning needs in genetic medicine and genetic counseling. Educational programs tailored to regional backgrounds, along with collaboration with clinical genetic specialists, especially certified genetic counselors, could contribute to ensuring equitable and high-quality genetic healthcare service.

## Highlights

• Nurses in a rural area of Japan had limited understanding of clinical genetics.
• Over 70 % of nurses expressed strong interest in learning about genetic medicine.
• Region-specific nursing education is key to ensure equitable genetic healthcare.

## 1 Introduction

Cancer genomic medicine has rapidly advanced with progress in cancer research and genomic technologies (Mano, 2020). Cancer genomic profiling (CGP) plays a central role in the cancer genomic medicine. CGP involves simultaneously analyzing multiple genes in mainly cancer tissues to select treatment drugs based on genomic information, providing treatment tailored to individual patients with cancers. In Japan, CGP became insurance-covered in June 2019, and by March 2025, 13 core hospitals and 32 regional hospitals, along with 234 affiliated hospitals, form a nationwide cancer genomic medicine network ensuring access across all prefectures (Mukai and Ueno, 2021).

CGP can also reveal germline pathogenic variants as secondary findings, which are important for diagnosing hereditary cancers and for enabling personalized risk assessment and preventive care for patients and their families (Minamoto et al., 2022). Appropriate genetic counseling is essential and is provided in collaboration with cancer care teams and certified genetic counselors (CGCs) (Best et al., 2022; Minamoto et al., 2022; Shimada et al., 2025). CGCs play key roles not only in genetic counseling but also in education (Abacan et al., 2019). Globally, the number of genetic counselors has grown to over 10,250 across nearly 50 countries by the end of 2022 (Ormond et al., 2024). However, disparities exist between urban and rural areas worldwide (Cohen and Nixon, 2016; Fogleman et al., 2019; Kaye et al., 2020; Triebold et al., 2021). Japan has only about 300 CGCs, with around 40 % working in urban areas, while many rural regions have few or none (Aizawa et al., 2021). In several countries or rural areas, hospital nurses fulfilled a critical role in basic counseling services after education in the basic clinical genetics, or standard methods for preliminary genetic counseling under appropriate collaboration with genetic specialists (Barr et al., 2018; Malherbe et al., 2017).

In Japan, the genetic nursing competencies required for general nurses were identified as “living support”，“psychological support”, “identification of the client's wishes”, and “collaborative work within a multidisciplinary team” (Arimori et al., 2007). However, genetic nursing education at undergraduate nursing programs has depended on each institution's curriculum, and continuing education opportunities remain limited (Funaki et al., 2023; Murakami et al., 2020; Tsuji et al., 2014). Several genetic nursing education programs have been developed for clinical nurses and nursing students (Asano et al., 2020; Murakami et al., 2020). Surveys of general hospital nurses have shown similar gaps in basic understanding but a high interest in learning more about genetic/genomic medicine (Araki et al., 2022; Sato et al., 2018; Satou et al., 2023). Similar issues have also been reported internationally, highlighting that integrating genetic/genomic medicine into nursing practice remains a global challenge (Calzone et al., 2018a; Calzone et al., 2024; Camak, 2016; Seven et al., 2015; Yeşilçinar et al., 2022). To empower all nurses to confidently engage in genetic medicine and to eliminate barriers to accessing genetic services, it is essential to identify and address nurses' genetics-related learning needs, especially in rural areas with limited CGC availability.

This study aimed to investigate hospital nurses regarding their experiences of care for patients with genetic diseases and their families, as well as their learning needs about clinical genetics or genetic counseling in Oita Prefecture, as a representative rural area of Japan.

## 2 Methods

### 2.1 Demographic and regional characteristics of Oita Prefecture

Oita Prefecture is situated in the northeastern coastal region of Kyushu Island. As of October 1, 2023, the population of Oita Prefecture was approximately 1,096,000 in an area covering 6340 km^2^. It includes 18 municipalities, including Oita City, which is the prefectural capital. In recent years, the population of Oita Prefecture has declined. The number of children under 15 years of age has decreased to 11.6 % of the population, while elderly residents over 65 years of age make up 34.2 %. These figures are slightly above the national average in Japan, where children account for 11.4 % of the population and the elderly account for 29.1 %. Consequently, Oita Prefecture reflects a microcosm of Japan's demographic trends, particularly those seen in rural areas.

### 2.2 Study participants

**Fig. 1:**
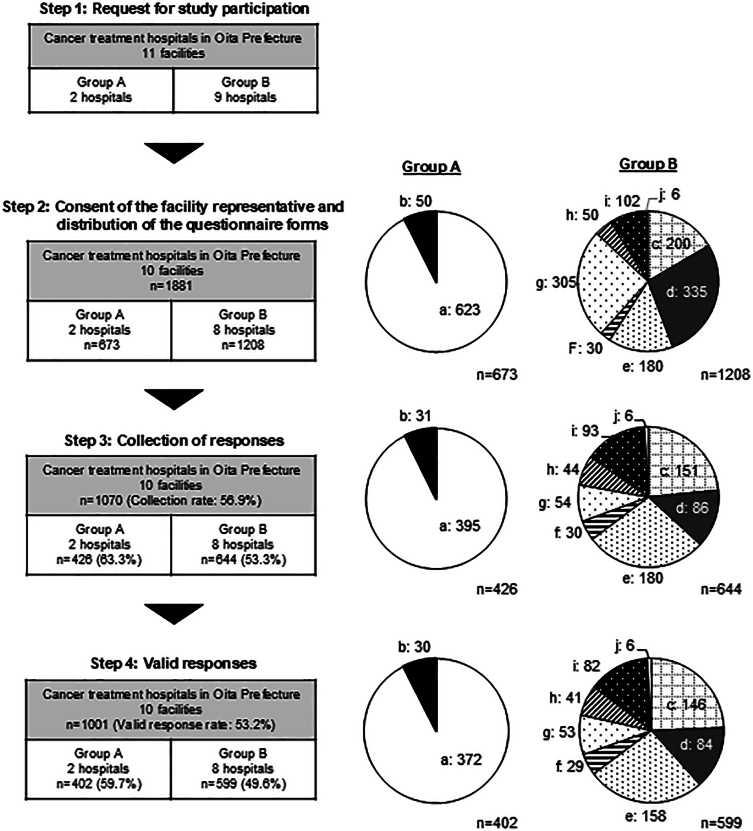
Recruitment flow of study participants in a cross-sectional study involving hospital nurses in Oita Prefecture, Japan (2021−2022). Note: Cancer treatment hospitals in Oita Prefecture: Group A: Central hospitals for cancer genomic medicine (Hospitals a, b), Group B: Other cancer treatment hospitals (Hospitals c, d, e, f, g, h, i, j).

The 11 institutions surveyed in this study consisted of all facilities in Oita Prefecture designated as cancer genomic medicine cooperative hospitals, regional cancer center hospitals, regional cancer hospitals, regional cancer care cooperative hospitals, or core hospitals within secondary medical care areas (Fig. 1). Research cooperation was requested from all 11 facilities, and signed consent forms were obtained only from 10. For comparative purposes, we categorized the 10 hospitals based on their role in cancer genomic medicine: two central hospitals actively providing cancer genomic medicine were designated as Group A, and eight other cancer treatment hospitals not providing such services were categorized as Group B. Group A consisted of two large hospitals with over 600 beds, one of which had an affiliated CGC. Group B included hospitals with 150 to over 400 beds, none of which had a CGC. In the regional healthcare system, Group B facilities serve as community medical centers, while patients requiring advanced care—including genetic counseling and treatment for hereditary diseases—are referred to Group A.

The participants consisted of registered nurses, midwives, and assistant nurses, excluding those primarily involved in nursing management. In this study, the term “nurses” was defined to consist of registered nurses, midwives, and assistant nurses. In Japan, registered nurses and midwives are nationally licensed, while assistant nurses are licensed by prefectures and work under supervision. Each facility's nursing director distributed an explanatory document, the questionnaire and a return envelope to the participants, totaling 1881 hospital nurses (Group A, *n* = 673; Group B, *n* = 1208) (Fig. 1).

### 2.3 Questionnaire survey

An anonymous self-administered questionnaire was administered and responses were collected via in-hospital collection boxes or mail. The survey period was October 11, 2021 to January 20, 2022. The questionnaire was developed based on previous studies (Nakagomi et al., 2009; Sato et al., 2018; Yokoyama et al., 2001) and included eight sections: participant demographics, interest in genetic medicine, understanding of genetic/genomic terms, perceptions of genetic diseases and information, experiences with genetic disease patients, experiences with genetic counseling, learning status and needs regarding genetic medicine, and their awareness of the Genetic Counseling Department at Oita University Hospital (Supplementary Table A.1).

### 2.4 Data analysis

The collected data were analyzed using descriptive statistics for Groups A and B, and the groups were compared by chi-square, Fisher's exact, residual analysis and Mann-Whitney *U* tests. Subgroup analysis on learning needs based on genetic counseling experience were also performed within each group. *P* values of <0.05 were considered to indicate statistical significance. SPSS (ver. 28) was used for all statistical analyses.

### 2.5 Ethical considerations statement and informed consent

This study was approved by the Ethics Board of Oita University on July 28, 2021 (approval number: 2151). The study adhered to the ethical guidelines for human subjects. All clinical research was conducted in accordance with the principles outlined in the 1975 Declaration of Helsinki, as revised in 2013. Institutional approval for participation in this study has been included in the explanatory document. Consent for participation was obtained by participants selecting “Agree” on the questionnaire and returning it.

## 3 Results

### 3.1 Study participants

A total of 1881 hospital nurses from 10 facilities were invited to participate in the study, with a valid response rate of 59.7 % (402 responses) from Group A and 49.6 % (599 responses) from Group B, resulting in a total of 1001 valid responses (Fig. 1). The demographic characteristics of study participants are presented in Table 1. The proportion of respondents in their 20s (39.8 %) and with less than 10 years of clinical experience (49.5 %) was relatively high in Group A. Group B had a high proportion of respondents in their 40s (37.6 %) and a significantly higher percentage of respondents with more than 10 years of clinical experience (72.3 %; *p* < 0.01). More than 95 % of the respondents in both groups were registered nurses. More respondents in Group A reported having had lectures on genetics during their basic nursing education at nursing colleges or vocational schools (47.3 %) than those in Group B (40.4 %). Approximately 40 % of the respondents in both groups expressed an interest in genetic medicine.

**Table 1:** Background of the study participants in a cross-sectional study of hospital nurses conducted in Oita Prefecture, Japan (2021–2022).

	**Facilities**				***p-*value**
	**Group A**		**Group B**		
	**n**	**%**	**n**	**%**	
Gender	*n* = 402		*n* = 599		
Male	29	7.2 %	56	9.3 %	0.23
Female	373	92.8 %	542	90.5 %	
Other^1)^	0	0 %	1	0.2 %	

Age (years)	n = 402		*n* = 598		
20–29	160	39.8 %	119	19.9 %	< 0.01⁎
30–39	106	26.4 %	169	28.3 %	
40–49	96	23.9 %	225	37.6 %	
50–59	35	8.7 %	69	11.5 %	
> 60	5	1.2 %	16	2.7 %	

Clinical experience (years)	n = 402		n = 599		
0–4	123	30.6 %	77	12.9 %	< 0.01⁎
5–9	76	18.9 %	89	14.9 %	
10–19	106	26.4 %	210	35.1 %	
20–29	76	18.9 %	171	28.5 %	
> 30	21	5.2 %	52	8.7 %	

Profession	n = 402		n = 599		
Registered nurse	387	96.3 %	583	97.3 %	0.20
Midwife	15	3.7 %	14	2.3 %	
Assistant nurse1)	0	0 %	2	0.3 %	
					
Attending lectures on genetics in the basic nursing education curriculum	n = 402		n = 599		
Yes	190	47.3 %	242	40.4 %	0.01⁎
No	36	9.0 %	86	14.4 %	
I don't remember	176	43.8 %	271	45.2 %	

Interest in genetic medicine2)	*n* = 400		n = 599		
Yes	158	39.5 %	246	41.1 %	0.62
No	242	60.5 %	353	58.9 %	

⁎ *p <* 0.05 by chi-square test. Underlined values indicate standardized residuals greater than 1.96, adjusted by a residual analysis.

1 Excluded from the statistical analysis by chi-square test.

2 The level of interest in genetic medicine was assessed on a 4-point scale: 'Very interested = 1,' 'Somewhat interested = 2,' 'Not very interested = 3,' and 'Not at all interested = 4.' Responses of 1 and 2 were categorized as 'Yes,' and 3 and 4 as 'No.'

### 3.2 Level of understanding of terminology in genetic/genomic medicine

**Fig. 2:**
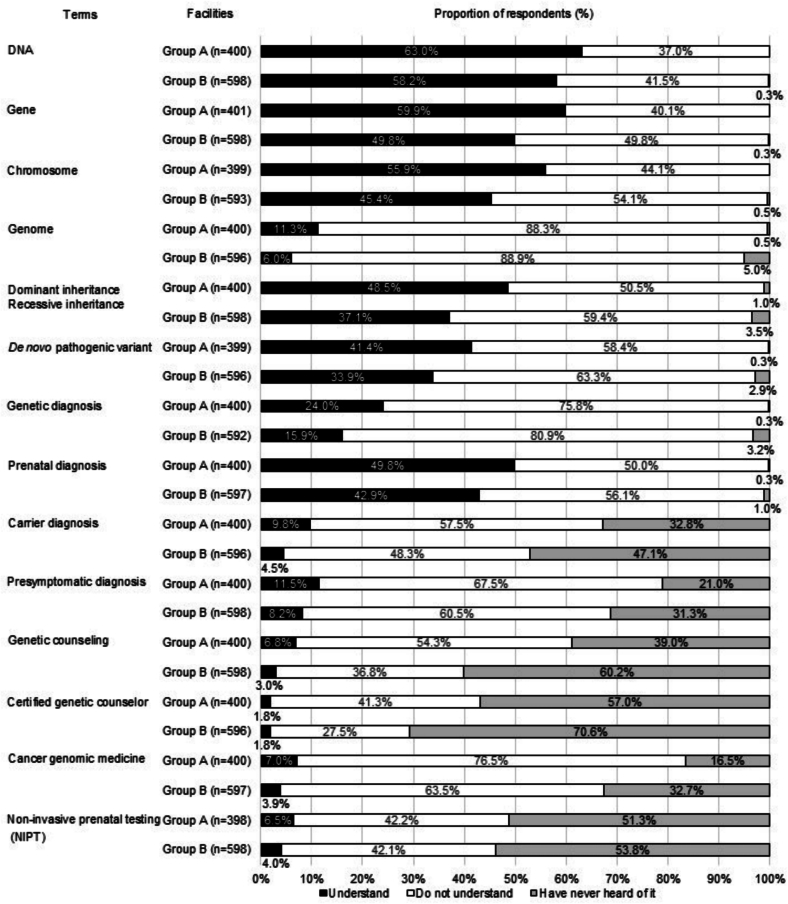
Comparison of self-assessment on understanding of terms related to genetic/genomic medicine between groups of hospital nurses in Oita Prefecture, Japan (2021–2022). Note: Group A: Central hospitals for cancer genomic medicine, Group B: Other cancer treatment hospitals. The degree of understanding of each term was assessed on a 5-point scale: ‘Understand and can explain to others = 1,’ ‘Largely understand = 2,’ ‘Somewhat understand but cannot explain well = 3,’ ‘Have heard of it but do not understand the meaning = 4,’ and ‘Have never heard of it = 5.’ Responses of 1 and 2 were categorized as ‘Understand,’ 3 and 4 as ‘Do not understand,’ and 5 as ‘Have never heard of it.’

The trends in understanding the 14 terminologies in genetic/genomic medicine are shown in Fig. 2. The terms that more than half of respondents of both groups answered they understood was “DNA” and “Gene,” and terms related to basic genetics, such as “Chromosome,” “Prenatal diagnosis,” “Dominant/Recessive inheritance,” or “*De novo* pathogenic variant,” were also answered relatively well understood. However, “Certified genetic counselor,” “Genetic counseling,” “Cancer genomic medicine,” “NIPT,” and “Carrier diagnosis,” all of which are related to clinical genetics/genomics, were poorly understood, as over 90 % of respondents answered that they had “never heard of” or that they “don't understand” the terms.

### 3.3 Experience in genetics-related training

Only a small proportion of respondents had participated in genetics-related training after being officially certified as nurses (1.5 % in Group A; 3.3 % in Group B) (Table 2). The most prevalent training in clinical genetics that very few respondents reported having received was provided by national-scale academic societies, nursing associations, or similar organizations. Only four respondents had participated in in-hospital training or local training in Oita Prefecture. The timing of this training was not investigated in this study.

**Table 2:** Participation in genetic medicine training since obtaining nursing qualification among hospital nurses in Oita Prefecture, Japan (2021–2022).

	**Facilities**				***p-*value**
	**Group A**		**Group B**		
	**n**	**%**	**n**	**%**	
Participation in genetic medicine training since obtaining nursing qualification	n = 402		n = 598		
Yes	6	1.5 %	20	3.3 %	0.05
No	359	89.3 %	504	84.3 %	
I don't remember	37	9.2 %	74	12.4 %	

Training format^1^, ^2^	n = 6		*n* = 18		
Training organized by national-scale academic societies, nursing associations, or similar organizations	4	66.7 %	10	55.6 %	
In-hospital training	1	16.7 %	1	5.6 %	
Local training in Oita Prefecture	0	0 %	2	11.1 %	
Training for certified nurses	0	0 %	3	16.7 %	
Cancer professional training plan-related seminars and e-learning	1	16.7 %	1	5.6 %	
Graduate lecture	1	16.7 %	0	0 %	
Others	0	0 %	2	11.1 %	

1 Multiple answers.

2 Answered only by respondents who selected “Yes” for participation in genetic medicine training since obtaining nursing qualification.

### 3.4 Awareness of the Genetic Counseling Department at Oita University Hospital

The awareness rate of the Genetic Counseling Department at Oita University Hospital was quite low; most respondents (60.8 % in Group A; 88.8 % in Group B) were not aware of it (Supplementary Table A.2).

### 3.5 Perceptions of genetic diseases, genetic information, and genetic counseling

**Supplementary Fig. A.1:**
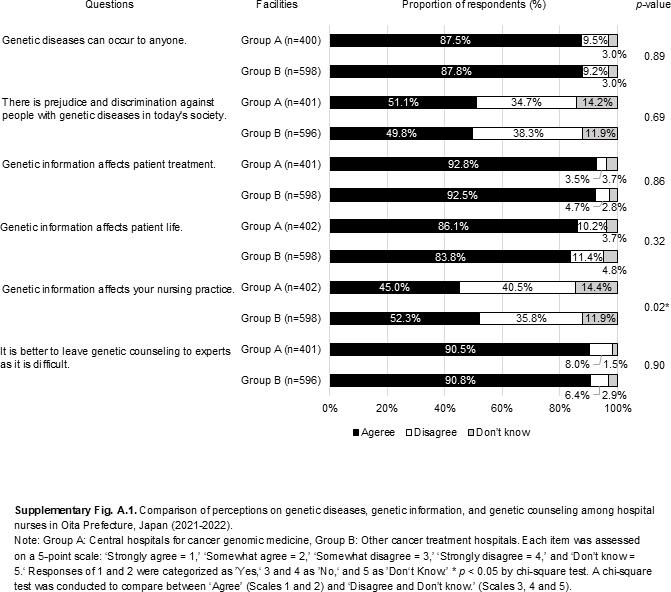
Comparison of perceptions on genetic diseases, genetic information, and genetic counseling among hospital nurses in Oita Prefecture, Japan (2021-2022).

The perceptions of genetic diseases, genetic information, and genetic counseling are shown in Supplementary Fig. A.1. Approximately 90 % of respondents answered that genetic diseases could occur in anyone, and recognized that genetic information affects patients' “treatment” and “life”, whereas only about 50 % answered that patients' genetic information affected their nursing practice. Over 90 % of respondents felt that genetic counseling was too difficult for non-specialists to perform.

### 3.6 Nursing experience for patients with genetic diseases

Group A respondents had a significantly higher percentage of nursing experience in relation to patients with genetic disorders (42.9 %) relative to Group B (30.1 %) (*p* < 0.01) (Supplementary Table A.3). The most frequent experience was caring for patients with neuromuscular diseases (approximately 20 %).

### 3.7 Nursing experiences with genetic counseling

Table 3 shows the clinical experience in relation to genetics-related questions asked by the patients and their families, and associated consultations. Only 86 of the 1001 respondents (8.6 %), 32 (8.0 %) in Group A, and 54 (9.1 %) in Group B had nursing experience with genetic counseling. The most common inquiry for these respondents was whether the patient's illness was a hereditary disease, followed by emotional distress and anxiety related to the hereditary nature of the disease. There were no statistically significant differences between the groups in any category. Only a few respondents referred patients to specialized facilities for genetic counseling or consulted with the genetic counselor or the medical social workers at the Cancer Consultation Support Center.

**Table 3:** Nurses' experiences and perceptions of genetics-related questions and consultations received from patients and their families in Oita Prefecture, Japan (2021–2022).

	**Facilities**						***p-*value**
	**Group A**			**Group B**			
	**n**	**%**	**rank**	**n**	**%**	**rank**	
Experiences of genetics-related questions and consultations	*n* = 399			*n* = 593			*p-*value^a^
Yes	32	8.0 %	–	54	9.1 %	–	0.55
No	367	92.0 %	–	539	90.9 %	–	

Contents of questions and consultations^1^, ^2^	*n* = 32			*n* = 54			*p-*value^b^
Whether the patient's illness is hereditary disease	25	78.1 %	(1)	42	77.8 %	(1)	1.00
Emotional distress and anxiety related to the hereditary nature of the disease	11	34.4 %	(2)	19	35.2 %	(2)	1.00
Information on symptoms, treatments, and management of the disease	7	21.9 %	(3)	15	27.8 %	(3)	0.62
Regarding prenatal diagnosis	6	18.8 %	(4)	10	18.5 %	(5)	1.00
Support (Medical, Welfare, Patient associations, etc.)	4	12.5 %	(5)	12	22.2 %	(4)	0.39
Seeking information on places to consult about genetic concerns	4	12.5 %	(5)	6	11.1 %	(7)	1.00
Whether and how to Inform patients, families, and relatives about genetic disease	3	9.4 %	(6)	9	16.7 %	(6)	0.52
Genetic testing (Definitive diagnosis, Presymptomatic diagnosis, Carrier testing)	3	9.4 %	(6)	5	9.3 %	(8)	1.00
Others	0	0 %	(7)	1	1.9 %	(9)	–

Respondings to genetics-related questions and consultations^1^, ^2^	n = 32			n = 54			*p-*value^b^
Answered with my knowledge	17	53.1 %	(1)	20	37.0 %	(4)	0.18
Actively listened	15	46.9 %	(2)	34	63.0 %	(1)	0.18
Consulted with physicians and then answered	15	46.9 %	(2)	28	51.9 %	(2)	0.82
Advised to consult directly with physicians	9	28.1 %	(3)	21	38.9 %	(3)	0.36
Consulted with nurses and then answered	5	15.6 %	(4)	8	14.8 %	(5)	1.00
Referred to a specialized facility for genetic counseling	3	9.4 %	(5)	4	7.4 %	(7)	1.00
Researched medical books or the internet by myself and then answered	1	3.1 %	(6)	6	11.1 %	(6)	0.25
Consulted with the genetic counselor	1	3.1 %	(6)	0	0 %	(10)	0.37
Consulted with the medical social workers at the Cancer Consultation Support Center	0	0 %	(7)	2	3.7 %	(9)	0.53
Responded “I don't know”	0	0 %	(7)	3	5.6 %	(8)	0.29

Presence of difficulties or concerns in answering to the genetics-related questions2)	n = 32			n = 54			*p-*value^b^
Yes	27	84.4 %	–	48	88.9 %	–	0.74
No	5	15.6 %	–	6	11.1 %	–	

Contents of difficulties and concerns^1^, ^3^	*n* = 27			*n* = 48			*p-*value^b^
Lack of specialized knowledge and information about genetic medicine	25	92.6 %	(1)	48	100 %	(1)	0.13
Difficulty in supporting the consultee with their decision-making process	18	66.7 %	(2)	18	37.5 %	(5)	0.02*
I was uncertain about how to address genetics-related questions as a nursing proffetional	14	51.9 %	(3)	33	68.8 %	(2)	0.21
Difficulty in mental support for the patient and their family	14	51.9 %	(3)	33	68.8 %	(2)	0.21
I was uncertain about referral to the appropriate professional	10	37.0 %	(4)	23	47.9 %	(3)	0.47
I was uncertain on how to communicate the potential risk of genetics to family members	6	22.2 %	(5)	21	43.8 %	(4)	0.08
I was uncertain how and when I should refer the patients and their family to specialized facilities for genetic counseling	5	18.5 %	(6)	11	22.9 %	(7)	0.77
I didn't have any medical staff nearby to ask for genetic concerns	4	14.8 %	(7)	12	25.0 %	(6)	0.39
It was difficult to mediate communication between the consultee and their family members	3	11.1 %	(8)	11	22.9 %	(7)	0.36

Self-evaluation of capability to provide answers for genetics-related questions and consultations2)	*n* = 31			*n* = 53			*p*-value^c^
Quite confident	0	0 %		0	0 %		0.93
Somewhat confident	1	3.2 %		3	5.7 %		
I can't confidently say either way	7	22.6 %		16	30.2 %		
I'm not sure how to answer properly	16	51.6 %		18	34.0 %		
I can't answer at all	7	22.6 %		16	30.2 %		

* *p* < 0.05, ^a^ chi-square test，^b^ Fisher‘s exact test，^c^ Mann-Whitney *U* test.

1 Multiple answers.

2 Answered only by respondents who selected “Yes” for experiences of genetics-related questions and consultations.

3 Answered only by respondents who selected “Yes” for presence of difficulties or concerns in answering to the genetics-related questions.

More than 80 % of the respondents with experience reported experiencing difficulties or concerns in providing answers to genetics-related questions from patients or their families (Table 3). The most common issue was a lack of specialized knowledge and information on genetic medicine, reported by 25 of 27 nurses (92.6 %) in Group A and all 48 nurses (100 %) in Group B. Only a few respondents in either group rated themselves as “able to provide appropriate answers to genetics-related questions.”

### 3.8 Learning needs in genetic medicine

**Fig. 3:**
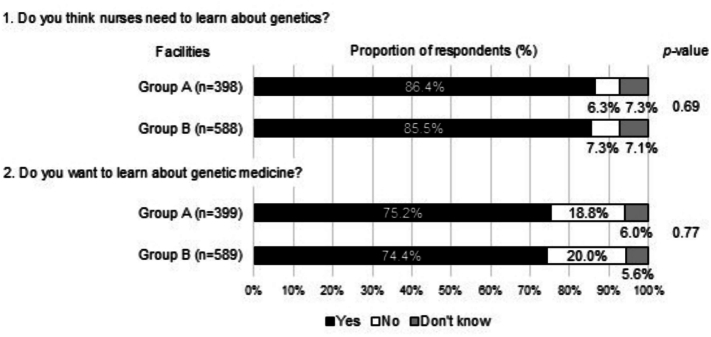
Comparison of perceived educational needs in relation to genetic medicine among hospital nurses in Oita Prefecture, Japan (2021–2022). Note: Group A: Central hospitals for cancer genomic medicine, Group B: Other cancer treatment hospitals. Each question was asked on a 5-point scale: ‘Strongly agree = 1,’ ‘Somewhat agree = 2,’ ‘Somewhat disagree = 3,’ ‘Strongly disagree = 4,’ and ‘Don't know = 5.’ Responses of 1 and 2 were categorized as ‘Yes,’ 3 and 4 as ‘No,’ and 5 as ‘Don't Know. * *p* < 0.05 by chi-square test. A chi-square test was conducted to compare between ‘Yes' (Scales 1 and 2) and ‘No and Don't know.’ (Scales 3, 4 and 5).

**Supplementary Fig. A.2:**
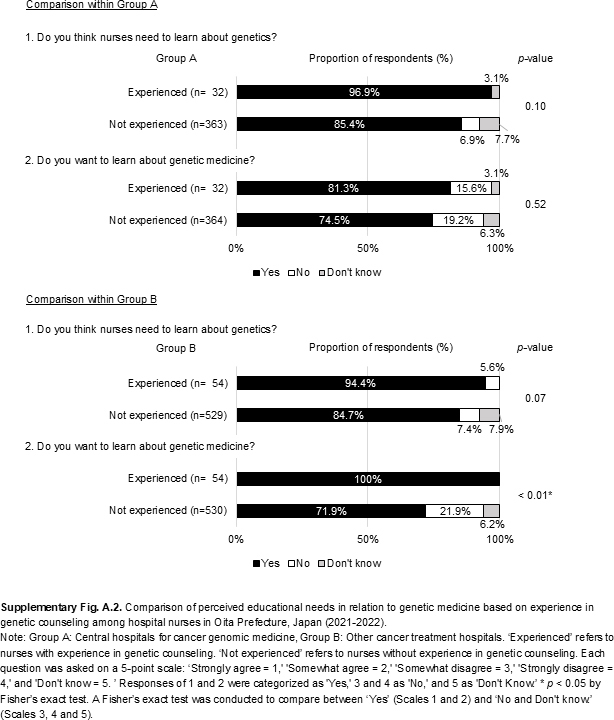
Comparison of perceived educational needs in relation to genetic medicine based on experience in genetic counseling among hospital nurses in Oita Prefecture, Japan (2021-2022).

**Supplementary Fig. A.3:**
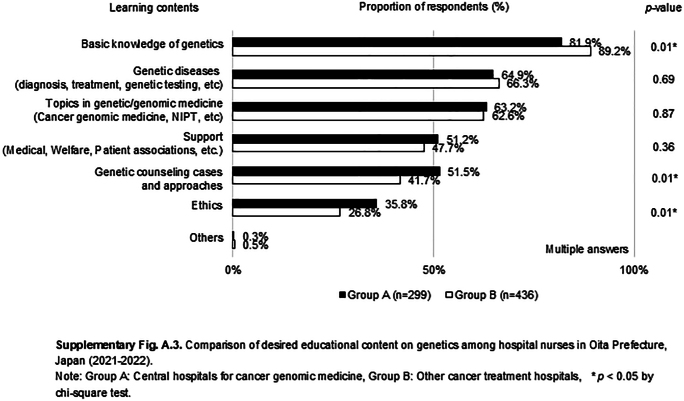
Comparison of desired educational content on genetics among hospital nurses in Oita Prefecture, Japan (2021-2022).

The genetic medicine learning needs are shown in Fig. 3. More than 80 % of the respondents in both groups answered that they needed to learn about genetics, and more than 70 % desired to learn about genetic medicine. All 54 respondents (100 %) in Group B with experience in genetic counseling expressed a desire to learn about genetic medicine, which was significantly higher than the 71.9 % of participants in Group B without experience in genetic counseling (*p* < 0.01) (Supplementary Fig. A.2). Supplementary Fig. A.3 shows the requested learning content. The most requested content from both groups was “basic knowledge of genetics.” Group A more frequently requested “genetic counseling cases and approaches” and “ethics” in comparison to Group B.

## 4 Discussion

This study identified the current state of understanding and learning needs regarding genetic medicine among hospital nurses in Oita Prefecture. Despite the growing importance of genetic/genomic medicine, particularly in cancer treatment, hospital nurses in rural areas have exhibited limited knowledge of genetic/genomic terminology and genetic counseling.

The low participation rate (< 5 %) in genetics-related training indicates insufficient educational opportunities locally. Previous studies reported somewhat higher training rates—13.2 % in the United States (Calzone et al., 2018b) and 24.3 % in Japan's urban and rural collaboration hospitals (Araki et al., 2022)—highlighting the regional gap. Furthermore, given that continuing genomics education for nurses in the United States has remained limited over the past two decades and that nurses' genomic competencies continue to be insufficient (Calzone et al., 2024; Thomas et al., 2023), it is reasonable to assume that the proportion of nurses who have received such training has not significantly changed in recent years. Basic genetic medicine education in local in-hospital training programs and advanced education from national conferences and education programs may help bridge the knowledge gap between urban and rural hospitals. Moreover, the significant interest in genetic medicine among rural nurses may suggest readiness to engage in learning when appropriate resources and training systems are available (Seven et al., 2015). We propose that CGCs and clinical genetic specialists at Oita University train nurses at each institution, who then act as core educators for their colleagues. We believe that this system can provide sustainable and effective genetic education in the rural areas in Japan.

Most hospital nurses in our study had not heard of specific terminology such as “CGC” or “genetic counseling.” The awareness of the Genetic Counseling Department at the Oita University Hospital was very low. These findings suggest that the concept of genetic counseling has not been sufficiently introduced among nursing professionals in Oita Prefecture. We believe that this result may be due to a lack of in-hospital education and training, or limited opportunities for inter-hospital collaboration in genetic counseling. Therefore, fostering associations between CGCs and nurses across local hospitals could lead to the provision of quality-controlled genetic counseling (Barr et al., 2018; Cohen and Nixon, 2016; Malherbe et al., 2017).

Regardless of whether nurses are involved in cancer genomic medicine, approximately 75 % of nurses in Oita Prefecture expressed a desire to learn about genetic medicine. This percentage is comparable to studies conducted in the United States (63.8 %), Istanbul, Turkey (70.3 %), as well as the rates among nurses at university hospitals located in urban areas of Japan (64.7 %), among nurses in cancer genomic medicine collaboration hospitals in urban and rural areas of Japan (71.5 %), and among nurses at core cancer genomic medicine hospitals in Kyushu, Japan (69.4 %) (Araki et al., 2022; Calzone et al., 2014; Sato et al., 2018; Satou et al., 2023; Seven et al., 2015). Hence, the learning needs related to genetic medicine among nurses in Oita Prefecture in Japan are similar to those in other countries and urban or rural areas. In South Africa, where there is a shortage of CGCs, the Medical Genetics Education Programme (MGEP) was developed to improve genetic healthcare for nurses in maternal and child health and women's healthcare (Malherbe et al., 2017). This program is based on remote education, self-administered home-learning, and face-to-face teaching, with instructions provided by genetic healthcare professionals, genetic counselors, and genetic nurse counselors. Over nine years, more than 1000 participants completed the program, leading to significant improvements in their genetics knowledge and skills in primary healthcare. In Japan, a competency-based introductory workshop using case studies has been reported as an effective initiative (Murakami et al., 2020). Many participants reported an increased understanding of the importance of genetics-informed nursing, along with improved knowledge and clinical confidence. Following these international education system, genetic/genomic medicine education programs in Oita Prefecture must include a multifaceted approach. Training programs should be carried out under combination of in-hospital training, outreach courses, open seminars, or online learning under the proper management. Through these processes, hospital nurses and clinical genetics professionals in Oita Prefecture will enhance both basic and applied competencies in genetic/genomic medicine and genetic counseling, while fostering collaboration.

This study has several limitations. The targeted subsets of hospital nurses were from 10 hospitals for cancer treatment in Oita Prefecture. Consequently, the findings did not necessarily reflect the awareness of genetic medicine and associated learning needs of all nurses in the region. Moreover, the study did not include hospital nurses working in urban areas, such as Tokyo, therefore, we could not directly compare urban and rural areas in Japan. The previously cited data on nursing education in the United States are over a decade old and may not accurately represent the current status of genomic training. Validation of the questionnaire was not conducted in this study; therefore, further research is required in this regard.

## 5 Conclusion

This study highlights the need to address the learning needs of hospital nurses regarding genetic medicine in rural areas as cancer genomic medicine has been widely deployed in Japan. It is essential to establish educational content on genetic/genomic medicine and to share comprehensive genetic counseling services, particularly in regions with limited access to specialists. We believe that this education system tailored to regional backgrounds, along with collaboration with clinical genetic specialists, especially CGCs, will enable all hospital nurses to enhance the quality of care for patients with cancer, genetic disorders, and their families. This educational system would further contribute to ensuring equitable and high-quality genetic healthcare service.

## CRediT authorship contribution statement

**Nobue Tsukatani:** Writing – review & editing, Writing – original draft, Project administration, Methodology, Investigation, Formal analysis, Conceptualization. **Yumi Shimada:** Writing – review & editing, Writing – original draft, Supervision, Investigation, Conceptualization. **Masanori Inoue:** Writing – review & editing, Writing – original draft, Supervision, Formal analysis, Conceptualization. **Akiko Hatanaka:** Writing – review & editing, Writing – original draft, Supervision, Investigation, Conceptualization. **Shizuyo Tominaga:** Writing – review & editing, Writing – original draft, Supervision, Investigation, Conceptualization. **Kenji Ihara:** Writing – review & editing, Writing – original draft, Project administration, Methodology, Investigation, Formal analysis, Conceptualization.

## Funding

This work was supported by 10.13039/501100001691JSPS KAKENHI Grant numbers [JP20K11071, JP24K14114].

## Declaration of competing interest

The authors declare that they have no known competing financial interests or personal relationships that could have appeared to influence the work reported in this paper.

## Data Availability

Data are available from the corresponding author upon reasonable request.

## Appendix A Supplementary data

Supplementary Table A.1 Survey questions and response options used for analyzing data collected from hospital nurses in Oita Prefecture, Japan (2021–2022). Supplementary material 1

Supplementary Table A.2 Nurses' awareness of the Genetic Counseling Department at Oita University Hospital in Oita Prefecture, Japan (2021-2022). Supplementary material 2
