# Supplementary material for: Learning needs and experiences of hospital nurses in genetic medicine in a rural area of Japan: A cross-sectional questionnaire survey in Oita prefecture
Source: Prev Med Rep. 2025 Sep 12;59:103239. doi: 10.1016/j.pmedr.2025.103239 (PMC12494937; doi:10.1016/j.pmedr.2025.103239)
Supplement: Supplementary file 1 [file mmc4.docx]

Supplementary Table A.1

Survey questions and response options used for analyzing data collected from hospital nurses in

The cover page of the questionnaire included definitions of 'Genetic disorder,' 'Genetic medicine,' and 'C

This table presents an English translation of the original Japanese questionnaire items used in the analysis.

Only the questions employed for data analysis are included and have been formatted as a list for clarity.

No.	Question
I. Informed consent *(Select one only)*	
Q1	Informed consent
II. Participant demographics *(Select one only)*	
Q2	Gender
Q3	Age
Q4	Clinical experience
Q5	Profession
III. Interest in genetic medicine *(Select one only)*	
Q6	How interested are you in genetic medicine?
IV. Level of understanding of terminology in genetic/genomic medicine *How much do you know about terms related to genetics/genomics? (Select one only)*	
Q7	DNA
Q8	Gene
Q9	Chromosome
Q10	Genome
Q11	Genetic diagnosis
Q12	Prenatal diagnosis
Q13	Carrier diagnosis
Q14	Presymptomatic diagnosis
Q15	Dominant inheritance / Recessive inheritance
Q16	*De novo* pathogenic variant
Q17	Genetic counseling
Q18	Certified genetic counselor
Q19	Cancer genomic medicine
Q20	Non-invasive prenatal testing (NIPT)
V. Perceptions of genetic diseases, genetic information, and genetic counseling *Please select the option that best reflects your view about genetics. (Select one only)*	
Q21	Genetic diseases can occur to anyone.
Q22	Genetic information affects patient treatment.
Q23	Genetic information affects patient life.
Q24	Genetic information affects your nursing practice.
Q25	It is better to leave genetic counseling to experts as it is difficult.
Q26	There is prejudice and discrimination against people with genetic diseases in today's society.
VI. Nursing experience for patients with genetic diseases	
Q27	Have you ever cared for patients with genetic disorders? *(Select one only)*
Q28	If you answered "1 = Yes" to Q27, please indicate the classification of genetic disorder patients you have cared for. *(Select all that apply)*
VII. Nursing experiences with genetic counseling	
Q29	Have you ever received genetics-related questions or requests for consultation from patients or their families? *(Select one only)*
