# Supplementary material for: Learning needs and experiences of hospital nurses in genetic medicine in a rural area of Japan: A cross-sectional questionnaire survey in Oita prefecture
Source: Prev Med Rep. 2025 Sep 12;59:103239. doi: 10.1016/j.pmedr.2025.103239 (PMC12494937; doi:10.1016/j.pmedr.2025.103239)
Supplement: Supplementary file 2 [file mmc5.docx]

Supplementary Table A.2

Nurses' awareness of the Genetic Counseling Department at Oita University Hospital in Oita Prefecture, Japan (2021-2022).

	**Facilities**				***p*-value**
	**Group A**		**Group B**		
	**n**	**%**	**n**	**%**	
	n=400		n=596		
I am very familiar with it	17	4.3%	2	0.3%	
I know some about it but not much	140	35.0%	65	10.9%	< 0.01*
I don't know it	243	60.8%	529	88.8%	

* $p < 0.05$ by chi-square test. Underlined values indicate standardized residuals greater than 1.96, adjusted by a residual analysis.
