# Supplementary material for: Learning needs and experiences of hospital nurses in genetic medicine in a rural area of Japan: A cross-sectional questionnaire survey in Oita prefecture
Source: Prev Med Rep. 2025 Sep 12;59:103239. doi: 10.1016/j.pmedr.2025.103239 (PMC12494937; doi:10.1016/j.pmedr.2025.103239)
Supplement: Supplementary file 3 [file mmc6.docx]

Supplementary Table A.3

Nursing experience related to patients with genetic disorders and classification of patients' diseases in Oita Prefecture, Japan (2021-2022).

	Facilities						*p*-value
	Group A			Group B			
	n	%	rank	n	%	rank	
Nursing experience of patients with genetic disorders	n=399			n=594			
Yes	171	42.9%	-	179	30.1%	-	< 0.01*
No	228	57.1%	-	415	69.9%	-	
Classification of patients' diseases[1)]	n=399			n=593			
Neuromuscular disorders	83	20.8%	(1)	113	19.1%	(1)	0.50
Bone and connective tissue disorders	73	18.3%	(2)	19	3.2%	(8)	< 0.01*
Cardiovascular diseases	56	14.0%	(3)	32	5.4%	(6)	< 0.01*
Chromosomal abnormalities	54	13.5%	(4)	74	12.5%	(2)	0.63
Hematologic and immunodeficiency disorders	49	12.3%	(5)	48	8.1%	(3)	0.03*
Hereditary tumors	34	8.5%	(6)	40	6.7%	(4)	0.30
Multiple congenital anomaly syndromes	33	8.3%	(7)	17	2.9%	(9)	< 0.01*
Metabolic and endocrine disorders	28	7.0%	(8)	34	5.7%	(5)	0.41
Renal and urologic disorders	25	6.3%	(9)	17	2.9%	(9)	0.01*
Dermatologic disorders	24	6.0%	(10)	21	3.5%	(7)	0.07
Others	5	1.3%	(11)	0	0%	(10)	-

* $p < 0.05$ by chi-square test.

[1)] Multiple answers
